# Geographic-genetic analysis of *Plasmodium falciparum* parasite populations from surveys of primary school children in Western Kenya

**DOI:** 10.12688/wellcomeopenres.11228.2

**Published:** 2017-09-05

**Authors:** Irene Omedo, Polycarp Mogeni, Kirk Rockett, Alice Kamau, Christina Hubbart, Anna Jeffreys, Lynette Isabella Ochola-Oyier, Etienne P. de Villiers, Caroline W. Gitonga, Abdisalan M. Noor, Robert W. Snow, Dominic Kwiatkowski, Philip Bejon

**Affiliations:** 1 KEMRI-Wellcome Trust Research Programme, Centre for Geographic Medicine Research-Coast, Kilifi, Kenya; 2 Wellcome Trust Centre for Human Genetics, University of Oxford, Oxford, OX3 7BN, UK; 3 Department of Public Health, Pwani University, Kilifi, Kenya; 4 Centre for Tropical Medicine and Global Health, University of Oxford, Oxford, OX3 7LJ, UK; 5 Spatial Health Metrics Group, Kenya Medical Research Institute-Wellcome Trust Research Programme, Nairobi, Kenya; 6 Wellcome Trust Sanger Institute, Cambridge, CB10 1SA, UK; 7 Centre for Clinical Vaccinology and Tropical Medicine, Oxford, OX3 7LJ, UK

**Keywords:** parasite mixing, genotyping, western Kenya, malaria, school surveys, spatio-temporal, micro-epidemiological, heterogeneity

## Abstract

**Background. **Malaria control, and finally malaria elimination, requires the identification and targeting of residual foci or hotspots of transmission. However, the level of parasite mixing within and between geographical locations is likely to impact the effectiveness and durability of control interventions and thus should be taken into consideration when developing control programs.

**Methods. **In order to determine the geographic-genetic patterns of *Plasmodium falciparum* parasite populations at a sub-national level in Kenya, we used the Sequenom platform to genotype 111 genome-wide distributed single nucleotide polymorphic (SNP) positions in 2486 isolates collected from children in 95 primary schools in western Kenya. We analysed these parasite genotypes for genetic structure using principal component analysis and assessed local and global clustering using statistical measures of spatial autocorrelation. We further examined the region for spatial barriers to parasite movement as well as directionality in the patterns of parasite movement.

**Results. **We found no evidence of population structure and little evidence of spatial autocorrelation of parasite genotypes (correlation coefficients <0.03 among parasite pairs in distance classes of 1km, 2km and 5km; p value<0.01). An analysis of the geographical distribution of allele frequencies showed weak evidence of variation in distribution of alleles, with clusters representing a higher than expected number of samples with the major allele being identified for 5 SNPs. Furthermore, we found no evidence of the existence of spatial barriers to parasite movement within the region, but observed directional movement of parasites among schools in two separate sections of the region studied.

**Conclusions.** Our findings illustrate a pattern of high parasite mixing within the study region. If this mixing is due to rapid gene flow, then “one-off” targeted interventions may not be currently effective at the sub-national scale in Western Kenya, due to the high parasite movement that is likely to lead to re-introduction of infection from surrounding regions. However repeated targeted interventions may reduce transmission in the surrounding regions.

## Introduction

Malaria incidence has markedly reduced in some parts of Africa ^1– 3^. In some instances, this has been associated with malaria control efforts ^4^, but has not been temporally associated with scaling-up malaria control in others ^5^. In either case, transmission becomes more heterogeneous ^2, 6– 12^, leading to the emergence of hotspots of symptomatic and asymptomatic infections that may be targeted as part of a malaria control strategy ^7, 9, 13– 17^. In order to predict whether targeting hotspots is potentially an effective way of interrupting transmission, we need to understand the spatial and temporal scales over which parasite mixing can be observed. Limited genetic differentiation between malaria parasites has been shown to occur on a regional scale in sub-Saharan Africa ^18– 20^, although we previously identified spatial structure at fine micro epidemiological scales within geographically defined regions in Kenya and The Gambia ^21^.

The level of parasite mixing is likely to impact the effectiveness of control interventions. In a recent randomized controlled trial of targeted integrated vector control in Rachuonyo south district in Western Kenya, an initial impact was seen within hotspot areas, but this did not reduce transmission outside the hotspot, and reductions within hotspots were not sustained ^9^. This may have been due to rapid mixing of parasites from areas outside the intervention zones. Furthermore, declining malaria transmission is associated with increased risk of imported cases of infection and disease from high transmission to low transmission regions, hampering elimination efforts in the low transmission regions ^22^, and risking the spread of drug resistant malaria in higher transmission regions ^23^. Thus, understanding parasite movement and gene flow will provide insights into novel, more targeted approaches to malaria elimination and combating the threats posed by re-introduction.

This study aimed to examine the geographic-genetic patterns of malaria parasite populations sampled at a sub-national level in Western Kenya, with a view of determining the extent of parasite mixing and genetic adaptation by the parasite population to its local environment. We genotyped 111 single nucleotide polymorphic (SNP) positions in *Plasmodium falciparum* isolates collected from children in 95 primary schools in two western Kenya provinces (Western and Nyanza) in order to analyse their genetic relatedness. We then examined the parasite population structure based on principal component analysis (PCA), and used measures of local and global spatial autocorrelation to test for geographical relatedness among parasite genotypes. We further analysed the geographical distribution of allele frequencies to identify evidence of genetic adaptation of parasite populations to their local environment, and examined the region for spatial barriers to parasite movement, as well as for patterns in the direction of movement in either north/south or east/west directions.

## Materials and methods

### Study population

**Figure 1.**
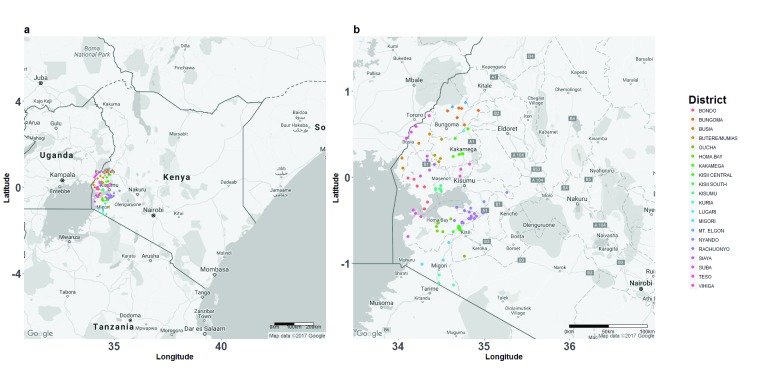
Spatial distribution of primary schools surveyed in a geographically defined region of western Kenya. 2486 *Plasmodium falciparum* positive samples were collected from children in 95 primary schools in 20 districts in this region. ( **A**) Each dot represents an individual school, colour-coded by the administrative district in which it is located. ( **B**) Map showing a close-up of the study region.

We sampled *P. falciparum* positive children from 95 primary schools in 20 districts in two provinces in Kenya (Western and Nyanza), located principally in the west of the country ( Figure 1). Kenya has recorded a decline in malaria transmission in the past decade ^2, 24, 25^, and current country-wide malaria prevalence is estimated at 8% ^26^. However, transmission is highly heterogeneous and the country can be divided into five malaria endemicity zones based on transmission intensity ^26^. The highest malaria transmission intensity is currently experienced in western Kenya and is characterized by stable, endemic transmission along the lowlands, and unstable, epidemic transmission within the highlands ^2, 26, 27^, and despite scale up of interventions, malaria transmission has remained high, or even increased in certain parts of this region ^28, 29^. *P. falciparum* is the main causative agent of malaria, and is transmitted by different *Anopheles* mosquito species in different parts of this region ^30, 31^.

### Ethics statement

During initial sample collection, consent for participation in the surveys was based on passive, opt-out consent by parents rather than written, opt-in consent, due to the routine, low-risk nature of the surveys that were carried out under the mandate of the Ministry of Public Health and Sanitation to conduct disease surveillance ^32^. Individual assent from the students was obtained before sample collection. Ethical approval for the current genotyping study was provided by Kenya Medical Research Institute (KEMRI) Ethical Review Committee (under SSC No. 2747) and study methods were carried out in accordance with approved guidelines.

### Sample collection and DNA extraction

Finger prick blood spots were collected during a parasitological survey of primary school children across the country between October 2008 and March 2010 ^32^. An in-depth description of sample collection has been published previously ^32, 33^. Briefly, 480 primary schools were surveyed and 49,975 samples collected, with a maximum of 110 children being randomly surveyed in each school. Samples were collected by spotting 3 separate drops of 200µl finger prick blood onto Whatman filter papers. These samples were then air dried and stored, with desiccant, at 4°C. Additionally, each child was tested for *P. falciparum* parasite infection using rapid diagnostic tests. During sample collection, geospatial coordinates for each school were recorded. 2486 of the samples collected from 95 schools in western Kenya were found to be parasite positive. DNA was extracted from these parasite-positive samples.

During DNA extraction, one of the blood spots was excised from each filter paper, cut into small pieces and placed into separate 1.5ml flip-top micro-centrifuge tubes (Eppendorf, Stevanage, UK). DNA was extracted using the QIAmp DNA investigator kit (Qiagen, UK), as per the manufacturer’s instructions on the Qiagen BioRobot. Picogreen (Fisher Scientific-UK Ltd, Loghborough, UK) was used to determine DNA concentration in each sample.

### SNP selection and genotyping

We typed 111 exonic SNP positions in 67 *P. falciparum* genes ( Dataset 1 and Supplementary Table 1) in each of the parasite positive samples. These SNPs were a subset of those previously used to test the sensitivity and specificity of a customised Illumina GoldenGate genotyping platform and to identify a molecular barcode for distinguishing parasites from different geographical regions ^19^. The 111 SNPs included those in well-known antigen-encoding genes, as well as housekeeping and hypothetical protein encoding genes. The SNPs were selected because they were genome-wide distributed, and polymorphic between at least two of three *P. falciparum* strains (3D7, HB3 and IT), which were selected because they are among the most studied and well characterised *P. falciparum* strains. Additionally, the SNPs had to be type-able on the genotyping platform (e.g. presence of a large enough conserved region around the SNP site that could be used to design locus specific primers for amplification). Genotyping was done on the Sequenom MassARRAY iPLEX platform ^34^. This mass spectrometry-based genotyping platform allows multiplexing of up to 40 SNPs in a single reaction well and is suitable for typing 10s – 100s of SNPs in 100s – 1000s of samples. 3D7 (PlasmoDB release 24) was used as a reference genome to design both locus specific PCR and iPLEX (SNP) extension primers using the Sequenom MassARRAY designer software (version 3.1). Locus-specific primers were pooled in a multiplexed PCR reaction and un-incorporated dNTPs were enzymatically dephosphorylated. The PCR products were then used in an iPLEX reaction where extension primers were bound immediately adjacent to target SNP sites and extended by a single nucleotide base into the SNP site using mass-modified dideoxynucleotides. Alleles were differentiated based on variations in their masses on the MALDI-TOF mass spectrometer.

### Sample and SNP cut-off selection

To determine Sequenom genotyping success rates, we aggregated genotype data for individual samples and SNPs and applied pass/fail criteria to genotyping. Our positive control criteria were to include samples where at least 60% of SNP typing was successful and, among these, to include SNPs that were successfully typed in at least 60% of all samples. The selection criterion for successful typing was based on individually defined SNP intensity values (R) ranging from 0 to 1. SNPs with intensity values <0.1 were considered low quality and were categorized as failed and excluded from further analyses. In addition, allelic intensity ratios (θ) nearing 0 or 1 were used to classify SNP positions as homozygous, and intensity ratios of intermediate values were used to classify SNPs as heterozygous, representing mixed parasite populations in a single sample. Where mixed parasite populations were identified, we took the dominant genotype forward for further analysis, as represented by the majority SNP calls. Applying these inclusion criteria, we restricted our analyses to 83 SNPs and 1809 samples collected from 88 schools in Western Kenya (Nyanza and Western provinces).

### Statistical analysis

Several statistical analyses were carried out either using R statistical software (version 3.3.1) ^35^ or SaTScan software (version 9.3) ^36^ ( Table 1).

**Table 1.** Statistical tests carried out on *P. falciparum* parasites collected from primary school children in Western Kenya.

Statistical analysis	Function
**Pairwise SNP and distance** **differences calculation**	Determining the number of SNP differences and distance differences between parasite pairs in the dataset.
**Minor allele frequency** **distribution**	Analysis of the frequency and distribution of the minor alleles in the population.
**Principal component analysis**	Detecting *P. falciparum* population structure at the sub-national level.
**Moran’s *I*** **spatial autocorrelation analysis**	Analysis of global spatial autocorrelation among parasite pairs at different geographical scales.
**Spatial scan statistics**	Identification of local, spatial clusters of parasite sub-populations.
**Logistic regression**	Analysis of the spatial pattern of distribution of allele frequencies among individual schools.
**Bernoulli regression**	Identification of spatial clusters of schools with similar frequencies at specific SNP loci.
**Raster analyses by pixels**	Detecting geographical regions that act as spatial barriers to parasite movement; Moran’s *I* analysis was then used to determine if pixels acting as barriers to parasite movement were spatially autocorrelated.
**Bearing regression analysis**	Analysing directionality in parasite movement within the region.

***Pairwise SNP and distance differences calculations.*** We computed parasite pairwise SNP and distance differences, comparing each parasite to every other parasite in the study population. For each parasite pair, we computed 1) the number of SNP differences at the 83 polymorphic positions analysed and 2) the distance between samples based on the geographical coordinates of the schools, taking 2.5km as an arbitrary distance between children attending the same school, assuming that on average schools were at least 5km apart, and taking 2.5km as the lower limit of detection of any two schools ( Dataset 2). SNP differences between parasite pairs were then aggregated at the school level to determine the mean number of SNP differences among parasites per school. Nucleotide diversity (π) in the parasite population was computed as the average number of SNP differences per site between two parasites in all parasite pairwise comparisons. We further analysed how the number of SNP differences among parasites varied with distance between schools.

***Analysis of parasite population genetics.*** Minor allele frequency distributions were computed for all 83 SNPs that had been successfully typed to determine the distribution of common and rare variants in the population. Population structuring was interrogated using principal component analysis (PCA) and computed based on singular value decomposition on a covariance matrix of pairwise SNP differences for all samples. SNPs that were included in the analysis, but were unsuccessfully typed in individual samples were replaced (in that sample) with the major allele in the population. Where there were mixed genotype infections in an individual sample, we took the major allele call to represent the dominant genotype at that position in that sample. PCA is a statistical technique widely used in analysis of genetic data where the number of genotypes interrogated is usually much higher than the number of samples analysed and is also useful when dealing with highly correlated data. The analysis transforms the original variables into new sets of variables that are linear combinations of the original variables, are uncorrelated and ordered based on the amount of variation in the original data that they explain. The first principal component explains most of the variation in the data, and subsequent components sequentially explain as much of the remaining variation as possible ^37^. Scores (values of the transformed variables that correspond to a specific data point) representing individual parasite genotypes were computed for the first 3 principal components. Since geospatial positioning information was collected at the school level and samples from the same school were assigned the same geographical coordinates, principal component (PC) scores were later aggregated at the school level, and the mean PC score for each school plotted on a map of the study region.

***Spatial autocorrelation analysis.*** To test the hypothesis that *P. falciparum* genetic structure has a spatial distribution component, we carried out an analysis of both global (Moran’s *I*) ^38^ and local (scan statistics) ^36^ spatial autocorrelation among parasite genotypes. Moran’s *I* simultaneously measures the correlation between feature attributes (parasite genotypes, represented here by the scores of the first 3 PCs) and locations (geospatial positioning of schools) to determine whether feature attributes are clustered, dispersed or randomly distributed in space. For each PC, Moran’s *I* correlation coefficients were computed for parasite pairs falling within 3 increasing distance classes of 1km, 2km and 5km. We used 100 bootstrap replicates to determine the statistical significance of the Moran’s *I* correlations observed for parasite pairs within each distance class.

Spatial scan statistics to detect statistically significant local clustering of genetically related parasites was carried out in SaTScan software (version 9.3) ^36^. We carried out this analysis separately for genotypes represented by each of the first three PCs. For each PC, scores representing individual parasite genotypes were imported into SaTScan, together with the spatial coordinates of sample collection. We used the latitude/longitude coordinates to specify feature (sample) locations and the PC scores to represent feature attributes (parasite genotypes), and ran a purely spatial, retrospective analysis based on a normal probability distribution model implemented in SaTScan software ^39^. We identified geographical regions with clusters containing parasite genotypes associated with high PC scores. In the normal probability distribution model, each observation or sample is associated with a single negative or positive continuous attribute (PC score) and the model uses a likelihood function based on the normal distribution. The spatial scan statistics employed here use a circular scanning window that is flexible both in location and size, with a radius that varies continuously from zero (including only a single sample location/school) to an upper limit set by the user (in this case 50% of the sample locations/schools). For each window location and size, a ratio of observed to expected PC scores was computed for samples found inside and outside the window. Clusters with high ratios were then noted and their statistical significance determined after accounting for multiple comparisons using random permutations. Shapefiles containing the spatial coordinates of statistically significant clusters were then imported into R and plotted on a map of western Kenya in order to identify locations of schools with genetically distinct parasite sub-populations.

***Spatial distribution of allele frequencies.*** We used a logistic regression model to examine geographic variations in allele frequencies. Each school was included in the model as a categorical independent variable, with the binary (1/0) outcome set as the presence or absence of a specific SNP in parasites within that school. We compared this model to a null model in a likelihood ratio test to test the goodness of fit of the model containing the SNPs and to identify those SNPs that showed statistically significant (p < 0.05) variations in frequency. To keep from inflating allele frequencies, SNPs that failed genotyping in individual samples were excluded from analysis. For those SNPs that showed a significant variation in frequency between schools based on the logistic regression model, we computed the frequency of each SNP per school and plotted these on a map of the region to visualize the distribution pattern of SNP frequencies in schools within the region.

To examine whether there were geographical clusters of schools with similar allele frequencies, we ran the Bernoulli model in SaTScan, including only SNPs that were statistically significant in the logistic regression model. At each SNP position, samples were coded with a 1 if they contained the major allele, and 0 if they contained the minor allele. The Bernoulli model analyses the distribution of cases (major allele) and controls (minor allele) and tests the null hypothesis that the distribution of cases and controls is random within the geographical area. The spatial scan statistics based on this model involves scanning the geographical space for regions with higher than expected number of cases. The statistical significance of identified clusters were computed as in the normal probability model above.

***Analysis of spatial barriers to parasite movement.*** To test for the presence of spatial barriers to parasite movement and mixing within the region, we used raster analysis implemented in R statistical software to divide the study area into 192 10km-by-10km grids/pixels. Each pixel was then scored with either 1 or 0 depending on whether or not a straight line linking a school pair crossed the boundaries of that pixel. This was done for all 192 pixels and all school pairs in the region. These scores were then included in a multivariable linear regression analysis to test how the presence of a specific pixel affected the nucleotide diversity of parasites in schools separated by that pixel. To determine the statistical significance of observed differences in nucleotide diversity, the analysis was bootstrapped based on 10,000 resampling steps. This bootstrap value was chosen to allow us to obtain more precise estimates. We included the coefficient estimates derived from the pixel analysis in a Moran’s *I* analysis to determine whether pixels acting as barriers to or gateways for parasite movement were spatially auto-correlated. Moran’s *I* analysis was computed for schools falling within a 10km distance class, representing the same spatial extent as that used to generate the pixels. We used 100 bootstrap resampling steps to determine statistical significance in the Moran’s *I* analysis.

We further carried out a regression analysis based on bearing to examine the parasite population for patterns of directional movement, either in the north/south or east/west directions. For each 10km-by-10km grid, school pairs that crossed its boundaries (scored as 1 in the previous pixel analysis) were selected. Each pair of schools was then individually coded as 1 if the absolute difference in latitude between them was greater than the absolute difference in longitude, indicating a north/south direction of movement, and coded as 0 if the reverse was true, indicating a west/east direction of movement. These new variables were then included in a multivariable linear regression analysis to test the effect of north/south or east/west directional movement on parasite genetic diversity. To determine the statistical significance of each pixel in acting as a force in directional movement, we ran a bootstrap analysis with 10,000 resampling steps. We then carried out Moran’s *I* analysis, using coefficient estimates derived from the bearings’ regression analysis as feature attributes, to examine the region for spatially auto-correlated directional movement. Moran’s *I* analysis was once again carried out for schools falling within 10km distance classes, and with 100 resampling steps to determine statistical significance.

## Results

### Sequenom assay performance

**Figure 2.**
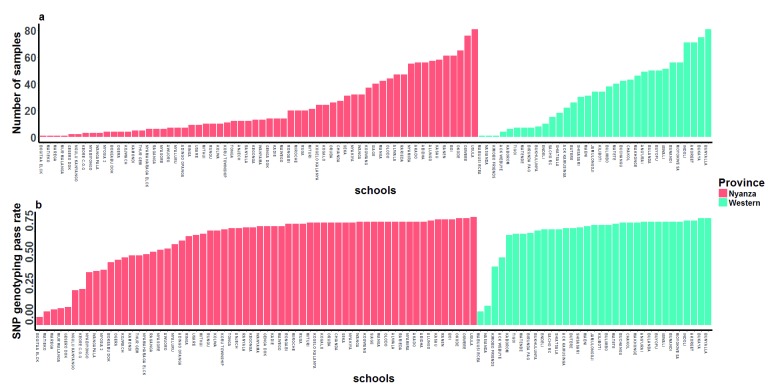
Distribution of *Plasmodium falciparum* positive samples and their associated genotyping success rates. ( **A**) 2486 samples were collected from 95 schools (33 in western province and 62 in Nyanza province) in western Kenya. The total number of samples varied from 1 to 81 per school. ( **B**) 111 single nucleotide polymorphic (SNP) positions were genotyped in all parasite positive samples. Mean genotyping success rates per school ranged from 6–86%.

We genotyped 111 genome-wide distributed exonic SNPs in 2486 *P. falciparum* positive samples collected from 95 primary schools in Western Kenya ( Figure 1 and Figure 2). 83 of the 111 SNPs were successfully typed in 1809 samples from 88 schools in Western Kenya (1097 from Nyanza province and 712 from Western province). Subsequent analyses were carried out on these 83 SNPs and 1809 samples. Variation in parasite prevalence was observed in the region, with areas north and west of Lake Victoria having higher infection prevalence than areas south and east of the lake ( Supplementary figure 1).

### *P. falciparum* population genetics

Analysis of the minor allele frequency distribution showed that most of the genotyped SNPs were present at medium to high frequencies in the parasite population, with 51 of the 83 successfully typed SNPs having minor allele frequencies of 5% or higher. We also observed a high level of within-population genetic diversity in the parasite population, with an average nucleotide diversity (π) of 0.184 per SNP site.

**Figure 3.**
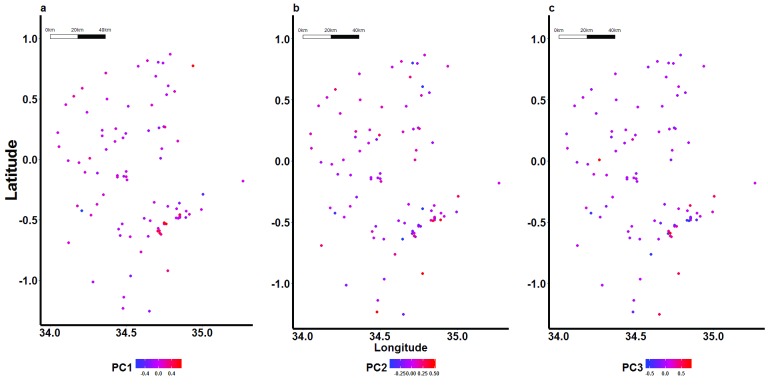
Spatial distribution of scores for the first 3 principal components (PCs) representing parasite genotypes. Geospatial positioning information was collected at the school level, thus PC scores (values of the transformed variables corresponding to a specific data point) were aggregated for all samples in an individual school. Here each dot represents a school, and has been colour-coded based on the mean genotype score of all parasite isolates collected in that school. Cumulatively, the first three PCs accounted for only 10.78% of the variation observed in the genotype data (PC1=3.74%, PC2=3.54%, PC3=3.5%).

Furthermore, using PCA, we found that the first three PCs cumulatively accounted for only 10.78% of the variation in the genotype data (PC1=3.74%, PC2=3.54%, PC3=3.5%), indicating high diversity among the parasites. At the sub-national level, we were unable to resolve the parasite population into distinct sub-populations based on PCA, and there was no difference in population structure in lower versus higher transmission intensity areas ( Figure 3).

### Spatial autocorrelation analysis

**Figure 4.**
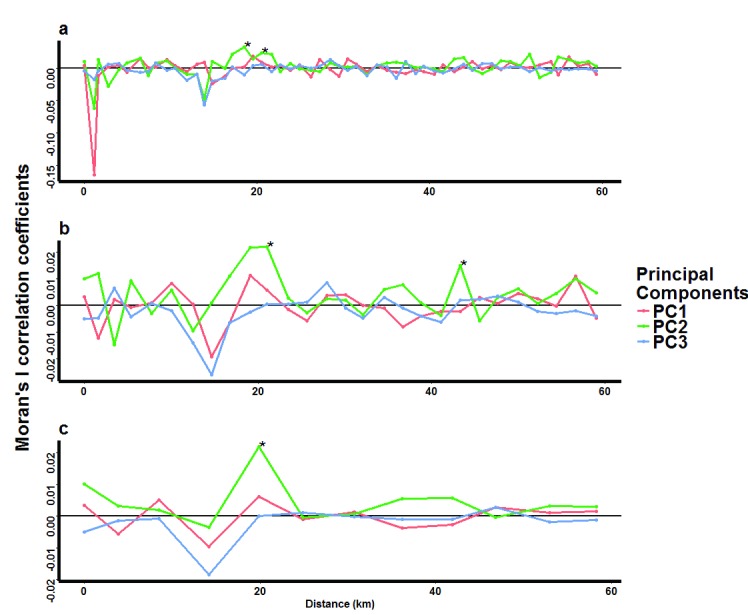
Moran’s *I* correlation coefficients describing the spatial autocorrelation of genotypes of *Plasmodium falciparum* parasite pairs. Spatial autocorrelation was tested separately for parasites grouped into three distance classes of **a**) 1km, **b**) 2km and **c**) 5km. Within each distance class, correlations were computed for each of the first 3 principal components (PCs). The asterisks represent those distances at which statistically significant (p<0.01) correlation coefficients were found for parasite pairs within each distance class, indicative of possible clustering of specific parasite genotypes.

To examine structure to the PC scores, we analysed both local (spatial scan statistics) and global (Moran’s *I*) spatial autocorrelation analysis. Spatial autocorrelation measures the extent to which geographical features and their associated data values are clustered, dispersed or randomly distributed in space. Moran’s *I* analysis showed no statistically significant trends of spatial autocorrelation among parasite pairs that were close to each other in any of the three distance classes (1km, 2km and 5km) analysed. We found significant autocorrelations (p<0.01) among parasites that were on average at least 20km apart in space ( Figure 4), but these autocorrelations were associated with very low correlation coefficients (< 0.03) and were not consistently seen in adjacent distance categories. Thus, the overall pattern seen from this analysis was that of little or no spatial autocorrelation in genotypes, even among parasite pairs that were very close to each other in space.

**Figure 5.**
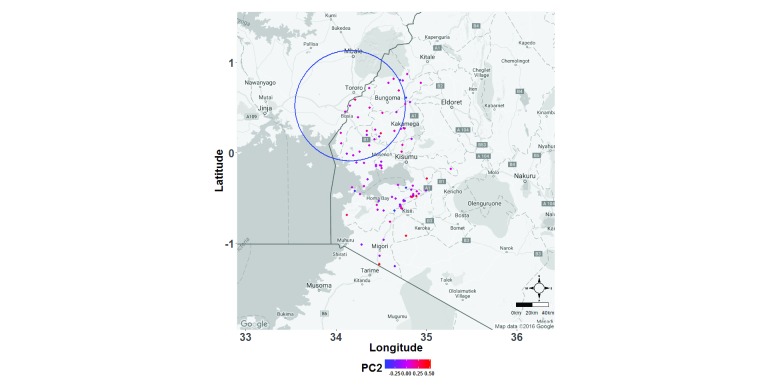
Spatial scan statistics to identify local spatially autocorrelated clusters of genetically distinct *Plasmodium falciparum* parasite sub-populations in western Kenya. Spatial scan statistics employing the use of multiple circular windows of varying sizes (ranging from covering only 1 sample up to 50% of the sample population) around samples geographically defined regions was used to compute the ratio between expected and observed number of genotypes within each window. Each window with higher than expected number of similar genotypes was noted down as a cluster, and its statistical significance determined after accounting for the multiple comparisons. Genotypes for individual parasites were assigned based on scores of the first 3 principal components. Here, each school is colour-coded based on the mean principal component score for all parasite genotypes found within it. One cluster of highly related parasite genotypes (blue circle) was identified when analysing the second principle component.

However, we identified one statistically significant (p=0.001) cluster based on PC2 when we analysed the data for local geographical clustering of distinct parasite genotypes using spatial scan statistics ( Figure 5). This cluster was relatively large, with a radius of 67.84km, and included 852 of the 1809 samples. We identified no significant clusters when we analysed the first and third PCs.

### Allele frequency distribution

We used a logistic regression model to examine the distribution of allele frequencies at each SNP position using log likelihood ratio testing for the effect of school. We identified 18 out of 83 SNPs that had statistically significant (p < 0.05) variations in frequencies among schools, although none of the SNPs were significant after Bonferroni adjustment for multiple testing.

We included these 18 SNPs in a spatial scan statistics analysis using the Bernoulli probability regression model and ran a purely spatial analysis to determine the geographic pattern of allele frequency distribution within the parasite population. For each SNP position, cases were represented by the major allele in the population while controls were represented by the minor allele. 5 of the 18 SNPs produced statistically significant geographical clusters containing schools with a higher than expected number of samples with the major allele ( Supplementary Table 2).

### Spatial variations in genetic differences between *P. falciparum* parasites

**Figure 6.**
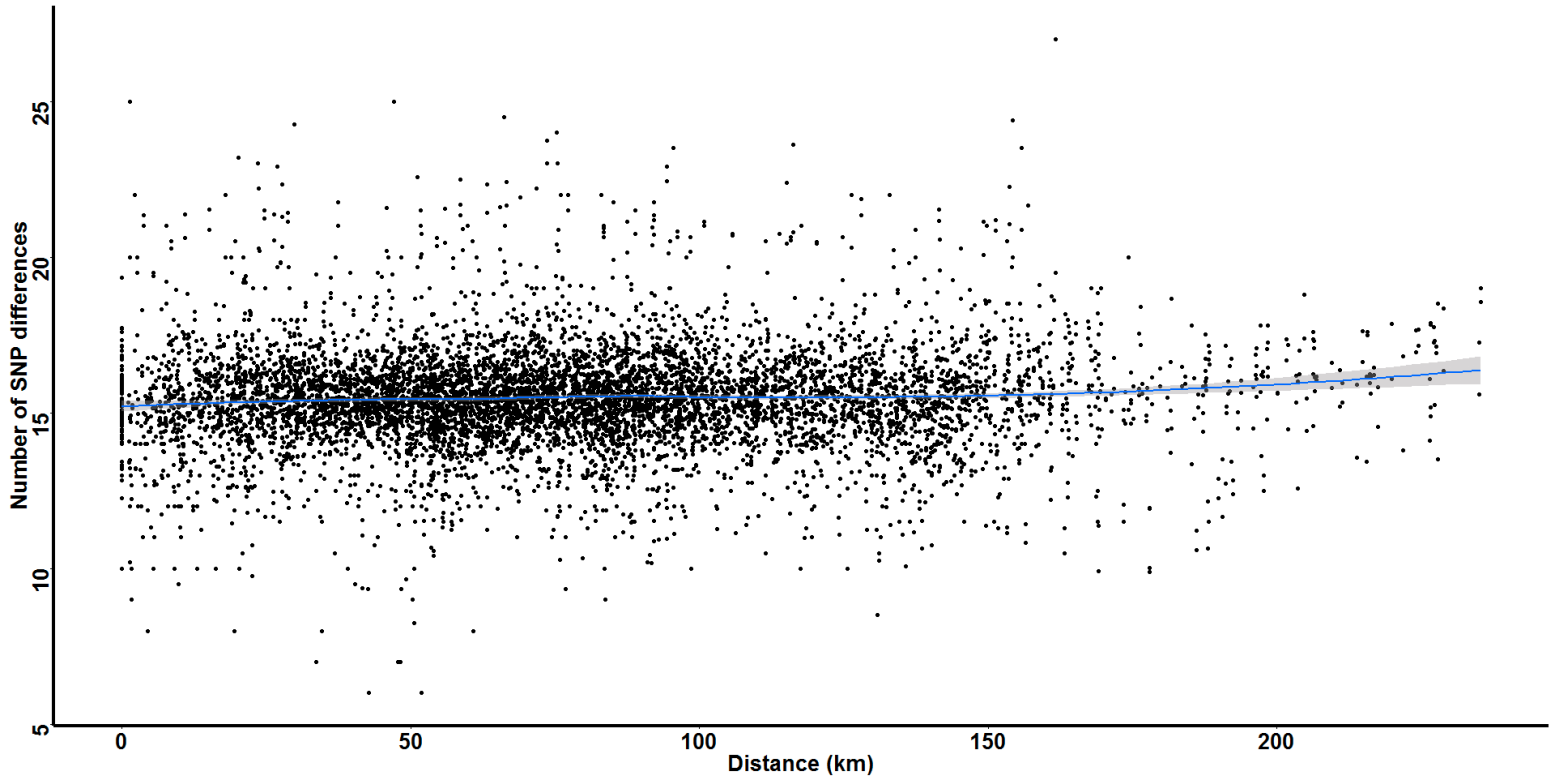
Variation in *Plasmodium falciparum* parasites’ genetic diversity over distance. Genetic diversity was defined as the average number of single nucleotide polymorphism (SNP) differences between parasites in each pairwise school comparison, and was plotted against the distance between the corresponding school pair. The blue line represents loss-fitted smoothing with 95% confidence intervals (grey area).

Using a linear regression model to examine the effect of distance on parasite genetic relatedness, we found that the number of SNP differences between parasite pairs was positively correlated with distance between the parasites (effect size = 1.85 × 10^ ^-3^) ( Figure 6). However, bootstrapping the analysis (to take into account the linked nature of pairwise observations) gave no statistically significant effects of distance on genetic relatedness (p=0.347; 95% CI = -0.012 – 0.017). These results provide no evidence for genetic isolation by distance in this parasite population.

### Spatial barriers to parasite movement and mixing

**Figure 7.**
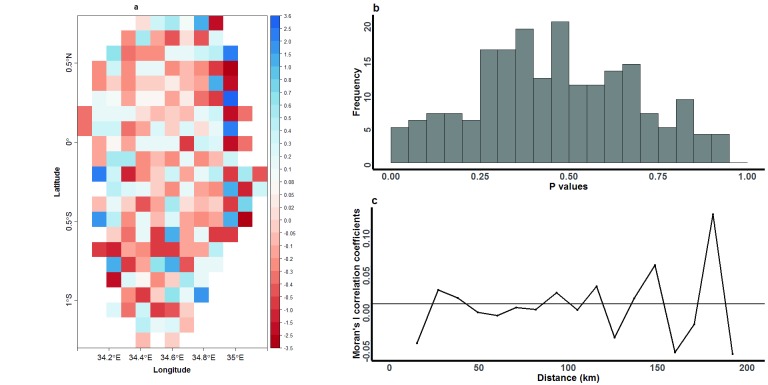
Raster analysis by pixels to examine the presence of spatial barriers to *Plasmodium falciparum* movement in a geographically defined region of western Kenya. ( **A**) Each pixel represents a 10km-by-10km area of the region, and is colour-coded based on the coefficient estimates derived from a linear regression analysis that was used to test the impact of each pixel in acting as either a barrier (blue pixels) or gateway (red pixels) to parasite movement with the region. No pixels were significant barriers or gateways to parasite movement after Bonferroni correction to account for multiple comparisons. ( **B**) Distribution of p values observed after bootstrapping the regression analysis (with 10,000 resampling steps) to determine the level of significance of pixels in acting as barriers to parasite movement. ( **C**) Moran’s *I* analysis describing the spatial autocorrelation between geographical locations of pixels and their associated coefficient estimates. Autocorrelation was calculated for parasites grouped in 10km distance bands, and the analysis was bootstrapped 100 times to determine significance.

We carried out raster analysis using 192 pixels to examine the study area for spatial barriers to parasite movement. Most of the pixels were found to have a non-significant influence on the number of SNP differences among parasites, and none were significant after correcting for multiple testing using the Bonferroni correction method ( Figure 7a). Furthermore, a histogram of p values showed a null (uniform) distribution ( Figure 7b), and an analysis of spatial relationships among pixels based on coefficient estimates derived from the pixel regression showed no evidence of autocorrelation among pixels acting as either barriers to or gateways for parasite movement ( Figure 7c).

**Figure 8.**
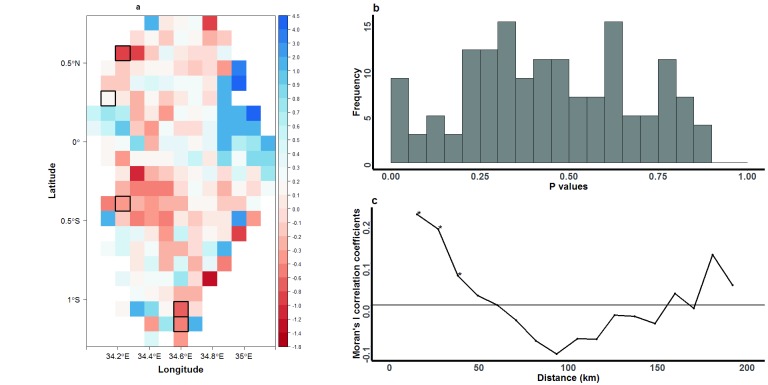
Raster analysis by pixels to examine patterns of north/south versus east/west directional movement of *Plasmodium falciparum* parasites in western Kenya. ( **A**) Each pixel represents a 10km-by-10km area of the region, and is colour-coded based on coefficient estimates describing the effect size of each pixel in influencing directional movement. Pixels that were statistically significant after correcting for multiple testing are highlighted with black borders. Grids were colour-coded to represent east/west (red) or north/south (blue) movement. ( **B**) Distribution of p values observed after bootstrapping the regression analysis (with 10,000 resampling steps) to determine the level of significance of pixels in influencing parasite directional movement. ( **C**) Moran’s *I* analysis to describe the spatial autocorrelation of movement within the region. The analysis was computed using geographical coordinates of individual pixels to represent feature locations and coefficient estimates derived from the bearing regression analysis to represent the associated feature values. Autocorrelation was computed for parasites grouped in 10km distance bands. Significant positive correlation coefficients (p<0.01; marked by asterisks) were observed for schools separated by up to 40 km within the 10km distance bands.

We further carried out raster analysis by pixels to examine the bearing (direction of movement) of parasites in either the east/west or north/south directions. Individually, most of the 192 pixels were not significant factors in determining directional movement of parasites over the region ( Figure 8a). However, some of the pixels were statistically significant (p<0.0003) in representing regions with greater east/west movement, even after accounting for multiple testing. When we included the regression coefficient estimates derived from analysis of bearing in a Moran’s *I* analysis to examine the parasite population for spatially auto-correlated direction of movement, we found evidence of statistically significant (p<0.01) autocorrelation for school pairs that were separated by up to 40km ( Figure 8c).

**Figure 9.**
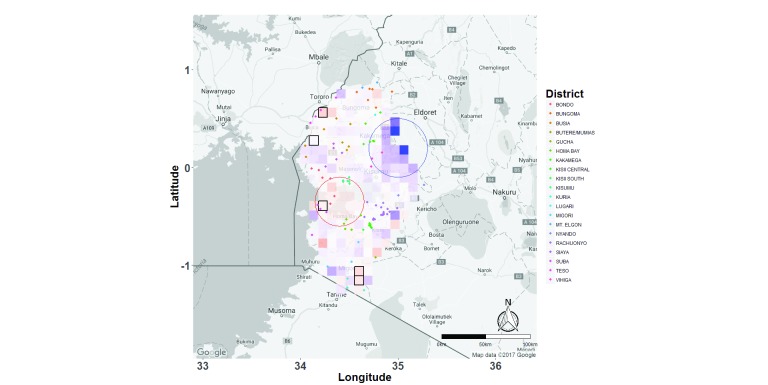
Map of the western Kenya study area with raster grids representing bearing analyses superimposed on top of it. Multivariable linear regression analysis was carried out to determine bearing (directionality of movement) of *Plasmodium falciparum* parasites among schools in the region. Grids are colour coded based on the coefficient estimates describing the effect size of that grid in influencing directional movement. Red represents east/west movement, while blue represents north/south movement. The grids with black borders represent those areas that were significant in east/west movement, even after Bonferroni-correction for multiple testing. The blue circle shows the region of the study site that had predominantly north/south movement, while the red circle represents that region that had predominantly east/west movement. Each dot represents a school, colour-coded based on the district in which the school is located.

Additionally, we identified two separate clusters of pixels within the region that showed patterns of specific directional movement, one in the north east (indicative of greater north/south movement) and another in the west (indicative of greater east/west movement) ( Figure 9).

Dataset 1: Genotyping results for 111 single nucleotide polymorphisms (SNPs) typed in 2486 Plasmodium falciparum samples collected from primary school children during a parasitological survey in western Kenya in 2009 and 2010. The columns contain the following information: sample_id, unique sample identifier; admin1, provincial location of school; district_name, district location of school; date_visit, date of sample collection; assay_code, name of assay; allele1 and allele2, alternative alleles at a specific SNP position; result, genotype call after processing; allele_ratio1, proportion of allele 1; allele_ratio2, proportion of allele 2; pass_fail, coding of SNP based on availability of valid genotype (pass=1) or lack of a valid genotype (fail=0). Geospatial data for individual school locations is considered sensitive data and therefore cannot be made open access. However, it can be accessed through a request to our data governance committee at dgc@kemri-wellcome.org. The criteria for such access is specified in detail in the data sharing guidelines under which the DGC operates, and relates to a) addressing health research, b)operating within the bounds of informed consent, c)complying with confidentiality procedures, d) mitigating potential harm to participants in research. Click here for additional data file. Copyright: © 2017 Omedo I et al. 2017

Dataset 2: Single nucleotide polymorphisms (SNPs) and distance differences between Plasmodium falciparum parasite pairs sampled during a parasitological survey of primary school children in western Kenya. Differences were computed for all parasite pairwise comparisons. Sample_id and sample_id_x are unique sample identifiers; snps represent the number of SNP differences between parasite pairs; distance represents geographical distance, in kilometres, between parasite pairs. Click here for additional data file. Copyright: © 2017 Omedo I et al. 2017

## Discussion

We have previously used SNP genotype data to examine the level of genetic relatedness among *P. falciparum* parasites on a micro-epidemiological scale within three regions with varying transmission intensities in Kenya and the Gambia, and found evidence of spatial sub-structure over short distances (i.e. <10km), despite a high level of parasite mixing ^21^. In the present analysis, we examined the level of parasite mixing at a sub-national scale in Western Kenya, using parasitological data from primary school surveys to describe the patterns of parasite mixing at a larger geographical scale.

We selected primary school children as the study population because they are easy to sample. Furthermore, infection diversity peaks at 3 – 14 years and then declines in older age groups in high transmission settings ^40– 42^; hence our study sample is likely to contain a diverse genetic pool representative of parasites circulating in the region. Sampling only asymptomatic infections in schools may not give the whole range of genetic diversity within the region, as one study identified specific polymorphisms in AMA1 that could have been more frequent in symptomatic infections compared with asymptomatic infections ^43^. Young children with symptomatic infections would be absent from school and away from the sampling frame. However, the sampling strategy was consistent across the different schools and the evidence of genomic variation in parasites according to clinical outcome is limited.

We found evidence of high genetic diversity in the Western Kenya parasite population, consistent with the high malaria transmission intensity experienced in this region ^2, 26, 27, 44^. Of the five malaria transmission zones in Kenya, Western Kenya currently experiences the highest transmission intensity ^24, 26^, despite efforts to scale up various control interventions, such as long lasting insecticide nets, indoor residual spraying and artemisinin combination therapy, in this region ^45– 47^.

Using PCA, we did not identify any genetic structure through inspection of the PC plots derived from SNP genotype data. This indicates an absence of discrete sub-populations within this *P. falciparum* parasite population, and is in agreement with our previous analysis of parasites from the same region ^21^, and with whole genome data from different African countries ^18, 20^. In South-East Asia, distinct sub-populations associated with antimalarial drug resistance have been described ^48^. Previous analyses of *P. falciparum* population structure in western Kenya have also shown high genetic diversity and little population differentiation in this parasite population ^49– 51^.

In contrast with a previous study of ours ^21^, analysis of trends in spatial relationships among parasite genotypes identified no significant autocorrelation using Moran’s *I* spatial autocorrelation analysis. Overall, the consistent pattern observed across all distance classes was that of no autocorrelation among parasites in schools at all distances, with occasional inconsistent associations that we considered likely to be spurious. Using the spatial scan statistics, we identified only a single cluster of genetically related parasites based on the second PC. This limited genetic clustering at both local and global scales, and weak evidence of genetic isolation by distance, are indicative of a parasite population that is well mixed at the sub-national geographical scale. This finding is in contrast to our micro-epidemiological study, which showed spatial structure to genetic relatedness over short distances ^21^.

In that previous study, however, we noted that the gradient between spatial separation and genetic relatedness was non-linear, and became less steep with distance such that past 10km there was little differentiation. This observation was hypothesized to be as a result of the rapid parasite movement and mixing observed within the study sites, with no geographical areas acting as spatial barriers to parasite movement, combined with a process operating at micro-geographical scales that results in a selection disadvantage to the autochthonous parasite population. In theory, the acquisition of parasite genotype-specific immunity or the impact of superinfection of incoming parasite types displacing existing parasites could meet these criteria. It is therefore consistent that we only identified a weak relationship in our study of schools where most pairs of schools were more than 10km apart.

We further examined the geographical distribution of allele frequencies for all 83 SNPs in our study population. Studies of allele frequency distribution have been used to determine parasite population structure and identify patterns of local adaptation of *P. falciparum* isolates ^52– 54^. Such local adaptation may be due to various selection pressures, including environmental pressure and immune selection, and may occur at individual, population or regional scales ^55, 56^. Of the 83 SNPs examined, we identified 18 SNPs that had statistically significant variations in allele frequencies among schools from the logistic regression analysis, although none of these 18 SNPs were significant after accounting for multiple testing. These findings suggest that although we were not sufficiently powered to distinguish any individual SNPs as likely to be significant beyond a Bonferroni correction, on the other hand the fact that 18 SNPs showed a value of p<0.05 when only 4 would be expected by chance suggests that there may be some genuine differences in frequency within the group of SNPs.

We reasoned that genuine geographical variation would be likely to show spatial clustering as well as variation by school, and so we measured the scan statistic for those showing significant variation among schools. 5 of the 18 SNPs that were identified showed local clustering among schools. However, these SNPs were not entirely private to a sub-population and occurred in schools inside and outside the clusters. This finding provides weak support for the existence of variable local genetic selection pressures in this parasite population. The identification of SNPs with significant geographical variation in allele frequencies could indicate adaptation of *P. falciparum* parasite populations to their local environment, or more likely may indicate a temporary expansion of a parasite sub-population with a particular SNP simply due to random genetic drift.

An extensive analysis of the study area for spatial barriers to parasite movement using 10km-by-10km sized pixels provided little evidence for the existence of geographical regions that act as barriers to parasite movement at the sub-national scale, and is in agreement with our previous study which did not identify any barriers to parasite movement at a micro-epidemiological scale ^21^.

This observation of free movement over the western Kenya region is supported by a previous analysis of mobile phone data that was used to analyse patterns of human movement within the country ^57^, and which showed substantial movement of people within this region, and further supports our observation of little or no barriers to parasite movement within the region. However, we also observed a cluster of pixels representing predominantly north/south movement in the north east and another cluster representing predominantly east/west movement in the west of the study area, and when we analysed the site for spatial autocorrelation in the directionality of pixels, we found statistically significant autocorrelation for school pairs separated by up to 40km. This means that pixels with greater east/west movement were more frequently found next to other pixels with greater east/west movement, and similarly, pixels with greater north/south movement were more frequently found next to pixels with greater north/south movement. Furthermore, some individual pixels showed statistically significant directionality that met Bonferroni-adjusted significance criteria. Although spatial autocorrelation of directionality might simply be because the same data (the same school pairs) cross pixels that are physically close to each other, our observation of two large clusters of pixels with distinct directional patterns of parasite movement is unlikely to have been an artefact of the same school pairs being analysed when all pixels in the clusters were considered, suggesting that we are able to detect specific migration pathways of parasites.

The findings in this study have several implications for the outcomes of malaria control programmes. Since we show that parasite populations mix to high degrees within the region, with little evidence of geographical clustering, one might conclude that interventions targeting smaller geographical areas within the region are likely to reduce the flow of parasites to regions beyond the targeted region. However, the high degree of parasite mixing also means that parasites move relatively freely within the region, and there is therefore a high likelihood of importation of infection from untargeted to targeted regions. This is strongly corroborated by evidence from a cluster-randomized controlled trial in a highland region of western Kenya that showed no impact in reducing transmission inside hotspots 16 weeks after applying interventions, possibly due to the importation of parasites from untargeted surrounding regions ^9^.

This study had some limitations. First, we cannot be definite about the time-scale over which gene flow has occurred. If the gene flow is rapid, this supports our conclusions regarding malaria control. On the other hand, it is possible that the well-mixed population emerged over a longer period of time and that gene flow, while resulting in complete mixing, could be less rapid, in which case targeted interventions would probably not have far reaching effects in the surrounding community. Our previous study showing spatial and temporal structure at a fine micro-epidemiological scale suggests rapid gene flow ^21^. In that study, we showed that parasite pairs taken from nearby homesteads had fewer SNP differences between them than parasite pairs that were further apart. However, over the period of a month this distance gradient was attenuated, and was gone by one year. However, more definitive work will require an in-depth analysis of whole genome data to identify haplotypes and rare variants in the population, and infer variation over time.

Second, geospatial coordinate data was collected for schools as opposed to individual homesteads, and hence genotype data was aggregated at this level. We were therefore unable to detect structure at micro-epidemiological scales. Third, we analysed only a small number of SNPs. This made it impossible to detect relatively rare private SNPs. It is likely that a larger set of genetic markers will be required to identify private SNPs and evidence of local parasite adaptation. SNPs in genes previously shown to be under selection in the parasite genome may additionally be analysed to determine whether population structure is observed based on local variations in selection pressure. Our previous study showed no population structure when SNPs in EBA175 and AMA1 were analysed ^21^. We therefore did not type and analyse separately SNPs in antigenic genes for the present study.

Fourth, we analysed genotype data collected from only one part of the country, thus we are unable to describe patterns of parasite flow across the country, or to generalize our findings to other geographical areas. Additional analyses of samples from other regions of the country that experience malaria transmission such as coast, eastern and north eastern provinces are recommended. Furthermore, over longer distances human movement becomes more important than mosquito movement in distributing parasites and therefore will need to be taken into consideration when analysing parasite genetic relatedness across large spatial scales. Information on travel distance can be obtained from travel history, or more objectively from mobile phone data, and can be used to track human movement between sources and sinks of parasite transmission. Concordance between spatial parasite genetic relatedness and human movement will further support our hypothesis of high parasite movement and mixing.

In conclusion, we have shown that parasites mix to high levels within the western Kenya region, with no evidence of parasite sub-populations and weak evidence of spatial autocorrelation of parasite genotypes at the local and global scales. We have also shown that directionality of parasite migration can be inferred based on genetic relatedness, and gene flow models, e.g. as implemented in Migrate-N software, can be used to determine the migration rates within the region, although such models are likely to prove more useful if distinct parasite populations exist and can be identified within the region.

## Data availability

The data referenced by this article are under copyright with the following copyright statement: Copyright: © 2017 Omedo I et al.

**Figshare: Dataset 1: Genotyping results for 111 single nucleotide polymorphisms (SNPs) typed in 2486 *Plasmodium falciparum* samples collected from primary school children during a parasitological survey in western Kenya in 2009 and 2010.**

Doi: http://dx.doi.org/10.6084/m9.figshare.4806619 ^58^

**Figshare: Dataset 2: Single nucleotide polymorphisms (SNPs) and distance differences between *Plasmodium falciparum* parasite pairs sampled during a parasitological survey of primary school children in western Kenya.**

Doi: http://dx.doi.org/10.6084/m9.figshare.4806631 ^59^

## Supplementary material

**Supplementary Table 1: Sequenom assay design information for *Plasmodium falciparum* samples collected from primary school children during a parasitological survey in Western Kenya.** Data includes the locus and IPLEX specific primers used in the Sequenom reaction to amplify and genotype SNPs of interest. gene product, gene product name; gene_symbol, gene name; assay_code, name of SNP assay; chr_valid, chromosome number; coord_valid, SNP position on chromosome; reference_allele, 3D7 reference allele; non_reference_allele, alternative allele; sequence, 3D7 reference sequence spanning the SNP site; first_pcrp, first PCR primer sequence; second_pcrp, second PCR primer sequence; extension_primer, IPLEX extension primer sequence; ext1_call, extended IPLEX primer; ext1_mass, Mass of the extended IPLEX primer; ext1_seq, sequence of extended IPLEX primer; ext2_call, IPLEX primer with alternative extended allele; ext2_mass, Mass of the extended IPLEX primer with alternative allele; ext2_seq, sequence of extended IPLEX primer with alternative allele.

**Supplementary Table 2: SNPs that showed significant clusters based on similarities in allele frequencies.** *Population size includes both cases (samples with the major allele) and controls (samples with the minor allele). Clusters were generated in SaTScan based on a Bernoulli probability model.

Supplementary figure 1: *P. falciparum* parasite prevalence in primary schools across Western Kenya. Each dot represents an individual school, colour-coded based on parasite prevalence (%). Parasite prevalence ranged from 0.9% – 62%.
