# Supplementary material for: Geographic-genetic analysis of Plasmodium falciparum parasite populations from surveys of primary school children in Western Kenya
Source: Wellcome Open Res. 2017 Sep 5;2:29. Originally published 2017 Apr 20. [Version 2] doi: 10.12688/wellcomeopenres.11228.2 (PMC5527688; doi:10.12688/wellcomeopenres.11228.2)
Supplement: Supplementary file 4 [file wellcomeopenres-2-13595-s0003.tgz › 4f31deb7-f909-4807-9f10-75880c5515ae.docx]

| SNP | Size of cluster (radius in km) | Population size* | No. of cases (samples with major allele) | P value |
| --- | --- | --- | --- | --- |
| 1 | 30.55 | 166 | 162 | 0.05 |
| 2 | 67.84 | 782 | 495 | 0.001 |
| 3 | 120.77 | 454 | 311 | 0.053 |
| 4 | 52.87 | 154 | 25 | 0.033 |
| 5 | 45.54 | 375 | 284 | 0.032 |

**Supplementary Table 2: SNPs that showed significant clusters based on similarities in allele frequencies.**

*Population size includes both cases (samples with the major allele) and controls (samples with the minor allele). Clusters were generated in SaTScan based on a Bernoulli probability model.
