# Supplementary figures and images for: Geographic-genetic analysis of Plasmodium falciparum parasite populations from surveys of primary school children in Western Kenya
Source: Wellcome Open Res. 2017 Sep 5;2:29. Originally published 2017 Apr 20. [Version 2] doi: 10.12688/wellcomeopenres.11228.2 (PMC5527688; doi:10.12688/wellcomeopenres.11228.2)

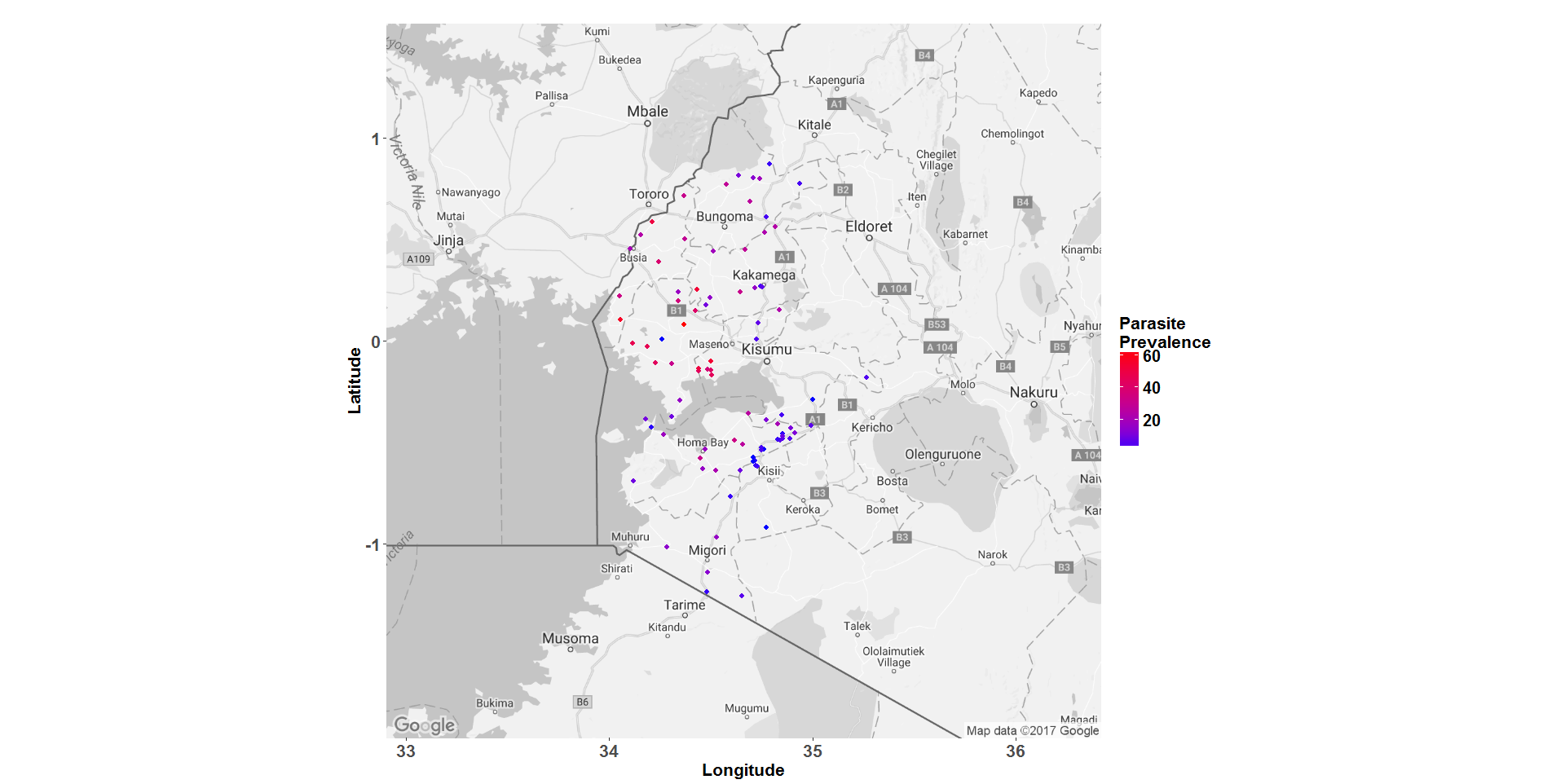

Supplement: Supplementary file 5 [file wellcomeopenres-2-13595-s0004.tgz › 880eedfd-8653-4102-b25a-60baae33985e.tif]
