# Supplementary material for: Geographic-genetic analysis of Plasmodium falciparum parasite populations from surveys of primary school children in Western Kenya
Source: Wellcome Open Res. 2017 Sep 5;2:29. Originally published 2017 Apr 20. [Version 2] doi: 10.12688/wellcomeopenres.11228.2 (PMC5527688; doi:10.12688/wellcomeopenres.11228.2)
Supplement: Peer review history

# Peer review: Referee response for version 2

**DOI:** 10.21956/wellcomeopenres.13595.r25684

**Published:** 2017-09-06

**Authors:** Lucy C. Okell

**Affiliations:** 1 MRC Centre for Outbreak Analysis & Modelling, Department of Infectious Disease Epidemiology, Imperial College London, London, UK

The authors have addressed my comments very thoroughly and I have no further additions.

I have read this submission. I believe that I have an appropriate level of expertise to confirm that it is of an acceptable scientific standard.

# Peer review: Referee response for version 1

**DOI:** 10.21956/wellcomeopenres.12114.r22048

**Published:** 2017-07-06

**Authors:** Lucy C. Okell

**Affiliations:** 1 MRC Centre for Outbreak Analysis & Modelling, Department of Infectious Disease Epidemiology, Imperial College London, London, UK

This is a very interesting and well-conducted analysis of spatial structure in parasite populations in Western Kenya, as assessed by detailed SNP barcoding of parasites. This will be useful information for malaria control programmes in order to understand the appropriate spatial scale at which to target interventions, as well as of much interest to researchers in this field. It builds on a number of studies which assessed spatial structure of parasites in other locations on a smaller scale, and adds useful information to these other studies.

 I have a few comments and suggestions:

- A number of different tests and analyses were conducted, which becomes slightly confusing. It would be helpful to have a list or table briefly indicating what each analysis is testing for.
- The authors mention this is an area of high transmission intensity – it would be interesting to additionally present a measure of malaria prevalence in the schools and roughly how much this varied across the area. Also, was there any difference in population structure in lower versus higher transmission areas? One might hypothesise that there could be.
- When an infection contained mixed parasite genotypes, the authors analysed only the dominant genotype. This is fine, but did they also consider using a method such as the REAL McCOIL ^1^? This is a statistical method for estimating SNP frequencies, using data from mixed infections, which avoids discarding the additional information available from minority genotypes. This might make quite a difference in this area of higher transmission, where presumably a fairly high proportion of infections contain more than one parasite population. I am not sure whether the sample sizes would be sufficient to run this method for each school, and thus from there complete the other analyses, but it would be worth investigating.
- In the methods section, could the authors add some more detail on this analysis, or provide a reference? “We then ran a purely spatial analysis based on a normal probability distribution model and located geographical regions with clusters of high PC scores.” I am not sure how easily others could reproduce it from its current description.
- The analysis looking at spatial barriers makes sense, but I wonder if the authors considered computing travel distance, and thus using more a priori information on potential barriers? Over these distances, presumably human movement may be more important than vector movement in distributing parasites. Although given the lack of spatial barriers identified by the pixel-based analysis, perhaps travel distance would be unlikely to show any different result.
- This section in the discussion: “Of the 83 SNPs examined, we identified 18 SNPs that had statistically significant variations in allele frequencies among schools from the logistic regression analysis. Although none of these 18 SNPs were significant after accounting for multiple testing, there was a clear excess of statistically significant SNPs, suggesting that the variations seen were not simply due to random chance.” I found rather confusing. If I read it correctly, it suggests that after multiple testing, the differences in frequencies were not significant, and could be due to random chance?
- I found this sentence very interesting: “In that previous study, however, we noted that the gradient between spatial separation and genetic relatedness was non-linear, and became less steep with distance such that past 10km there was little differentiation.”

           Could the authors elaborate on possible hypotheses for this observation? (or summarise from the previous paper if already included there). 

I have read this submission. I believe that I have an appropriate level of expertise to confirm that it is of an acceptable scientific standard.

## References

[1] ChangHHWorbyCJYekaANankabirwaJKamyaMRStaedkeSGDorseyGMurphyMNeafseyDEJeffreysAEHubbartCRockettKAAmatoRKwiatkowskiDPBuckeeCOGreenhouseB: THE REAL McCOIL: A method for the concurrent estimation of the complexity of infection and SNP allele frequency for malaria parasites. *PLoS Comput Biol*.2017;13(1) : 10.1371/journal.pcbi.1005348 e1005348 10.1371/journal.pcbi.1005348 28125584PMC5300274

# Response

**Published:** 2017-08-29

**Authors:** Irene Omedo

**Affiliations:** KEMRI- Wellcome Trust Research Programme, Kenya

We would like to thank you for reviewing our work and proving constructive advice on how to improve it as well as future studies. We have addressed your comments as shown below (in bold).

A number of different tests and analyses were conducted, which becomes slightly confusing. It would be helpful to have a list or table briefly indicating what each analysis is testing for.

**A table showing the statistical tests and their functions has been added under the “statistical analysis” heading in the methods section.**

The authors mention this is an area of high transmission intensity – it would be interesting to additionally present a measure of malaria prevalence in the schools and roughly how much this varied across the area. Also, was there any difference in population structure in lower versus higher transmission areas? One might hypothesise that there could be.

**A map of the study region showing parasite prevalence for each school has been added as supplementary figure 1. The following accompanying text has been added to explain the observation from the map.**

****

**“Variation in parasite prevalence was observed in the region, with areas north and west of Lake Victoria having higher infection prevalence than areas south and east of the lake (Supplementary Figure 1).”**

**No differences in population structure were observed in lower versus higher transmission areas, and a statement to this effect has been included in the results section under ‘P. falciparum population genetics’:**

****

**“At the sub-national level, we were unable to resolve the parasite population into distinct sub-populations based on PCA, and there was no difference in population structure in lower versus higher transmission intensity areas.”**

When an infection contained mixed parasite genotypes, the authors analysed only the dominant genotype. This is fine, but did they also consider using a method such as the REAL McCOIL ^1^? This is a statistical method for estimating SNP frequencies, using data from mixed infections, which avoids discarding the additional information available from minority genotypes. This might make quite a difference in this area of higher transmission, where presumably a fairly high proportion of infections contain more than one parasite population. I am not sure whether the sample sizes would be sufficient to run this method for each school, and thus from there complete the other analyses, but it would be worth investigating.

**Although THE REAL McCOIL seems to be robust at estimating complexity of infections and allele frequencies in samples with mixed infections, its focused mainly on determining SNP frequencies. For this reason, THE REAL McCOIL requires parasites to be initially assigned into distinct populations. We do not believe that this statistical method is appropriate in our case because we wanted to focus our analyses on individual parasite genotypes and their relatedness in pairwise analyses, to determine whether we can use genetic relatedness at this level to identify distinct parasite populations, and then use measures of spatial autocorrelation to identify the location and size of any parasite sub-populations.**

In the methods section, could the authors add some more detail on this analysis, or provide a reference? “We then ran a purely spatial analysis based on a normal probability distribution model and located geographical regions with clusters of high PC scores.” I am not sure how easily others could reproduce it from its current description.

**Additional information has been added to better explain the analysis. A reference has also been added. The additional text reads as follows:**

****

**“We used the latitude/longitude coordinates to specify feature (sample) locations and the PC scores to represent feature attributes (parasite genotypes), and ran a purely spatial, retrospective analysis based on a normal probability distribution model implemented in SaTScan software ^1^. We identified geographical regions with clusters containing parasite genotypes associated with high PC scores.”**

The analysis looking at spatial barriers makes sense, but I wonder if the authors considered computing travel distance, and thus using more a priori information on potential barriers? Over these distances, presumably human movement may be more important than vector movement in distributing parasites. Although given the lack of spatial barriers identified by the pixel-based analysis, perhaps travel distance would be unlikely to show any different result.

**Travel distance will add more information and help to draw more concrete conclusions. Such information can be derived from travel history or mobile phone data. However, we did not have this data at the time of analysis and thus did not include it. We have however, recommended this as a logical next step in any future analysis of parasite gene flow when using genetic data to analyse parasite movement. We have included a sentence in the discussion recommending this that reads as follows:**

**“Additional analyses of samples from other regions of the country that experience malaria transmission such as coast, eastern and north eastern provinces are recommended. Furthermore, over longer distances human movement becomes more important than mosquito movement in distributing parasites and therefore will need to be taken into consideration when analysing parasite genetic relatedness across large spatial scales. Information on travel distance can be obtained from travel history, or more objectively from mobile phone data, and can be used to track human movement between sources and sinks of parasite transmission. Concordance between spatial parasite genetic relatedness and human movement will further support our hypothesis of high parasite movement and mixing.”**

This section in the discussion: “Of the 83 SNPs examined, we identified 18 SNPs that had statistically significant variations in allele frequencies among schools from the logistic regression analysis. Although none of these 18 SNPs were significant after accounting for multiple testing, there was a clear excess of statistically significant SNPs, suggesting that the variations seen were not simply due to random chance.” I found rather confusing. If I read it correctly, it suggests that after multiple testing, the differences in frequencies were not significant, and could be due to random chance?

**The statement has been clarified as follows:**

****

**"Of the 83 SNPs examined, we identified 18 SNPs that had statistically significant variations in allele frequencies among schools from the logistic regression analysis, although none of these 18 SNPs were significant after accounting for multiple testing. These findings suggest that although we were not sufficiently powered to distinguish any individual SNPs as likely to be significant beyond a Bonferroni correction, on the other hand the fact that 18 SNPs showed a value of p<0.05 when only 4 would be expected by chance suggests that there may be some genuine differences in frequency within the group of SNPs."**

I found this sentence very interesting: “In that previous study, however, we noted that the gradient between spatial separation and genetic relatedness was non-linear, and became less steep with distance such that past 10km there was little differentiation.”

Could the authors elaborate on possible hypotheses for this observation? (or summarise from the previous paper if already included there). 

**The following statement has been added to bring out the hypothesis of high parasite mixing drawn from the previous study. That study has also been referenced:**

****

**"This observation was hypothesized to be as a result of the rapid parasite movement and mixing observed within the study sites, with no geographical areas acting as spatial barriers to parasite movement, combined with a process operating at micro-geographical scales that results in a selection disadvantage to the autochthonous parasite population. In theory, the acquisition of parasite genotype-specific immunity or the impact of superinfection of incoming parasite types displacing existing parasites could meet these criteria."**

# Peer review: Referee response for version 1

**DOI:** 10.21956/wellcomeopenres.12114.r23564

**Published:** 2017-06-19

**Authors:** Liwang Cui

**Affiliations:** 1 Department of Entomology, Pennsylvania State University, University Park, PA, USA

This study analyzed the genetic structure of 2486 Plasmodium falciparum parasites collected from children in 95 primary schools in western Kenya. Using genotypes of 83 SNPs in 1809 samples collected from 88 schools, the authors did not identify clear genetic structuring of parasite populations using different spatial analysis methods. This study followed an earlier example, which compared parasite populations on a larger geographical scale. Similarly, no spatial barriers of parasite movement were identified, and two regions showed evidence of directional parasite movement. This is another nice piece of work, which provides useful information for the local malaria control programs. 

 Comments:

1. The authors may want to see whether separate analyses with antigenic SNPs (which may be under balancing selection) and other SNPs will reach similar conclusions.
2. The author may use a wider range colour coding for Figures 3 and 5 – the current colour scheme is difficult to see the differences. 
3. Additional methods such as Migrate-n may provide further evidence on the directionality of parasite movements.

I have read this submission. I believe that I have an appropriate level of expertise to confirm that it is of an acceptable scientific standard.

# Response

**Published:** 2017-08-29

**Authors:** Irene Omedo

**Affiliations:** KEMRI- Wellcome Trust Research Programme, Kenya

We would like to thank you for reviewing our work and proving constructive advice on how to improve it as well as future studies. We have addressed your comments as shown below (in bold).

The authors may want to see whether separate analyses with antigenic SNPs (which may be under balancing selection) and other SNPs will reach similar conclusions.

**The following statement has been added in the discussion section to address this point:**

**“SNPs in genes previously shown to be under selection in the parasite genome may additionally be analysed to determine whether population structure is observed based on local variations in selection pressure. Our previous study showed no population structure when SNPs in EBA175 and AMA1 were analysed, we therefore did not type and analyse separately SNPs in antigenic genes for the present study.”**

The author may use a wider range colour coding for Figures 3 and 5 – the current colour scheme is difficult to see the differences.

**Due to the high level of similarity among the parasite genotypes analysed, most of the PC scores are quite similar in value, and this would be reflected in any continuous colour scheme chosen. Furthermore, we plotted the mean PC score per school, which further reduced the variability of the individual data points. The colour range shown in the figures is therefore more a factor or the PC values, rather than the specific colours used.**

Additional methods such as Migrate-n may provide further evidence on the directionality of parasite movements.

**Although Migrate-N is a useful software for inferring population size and migration rates, it seems to work best when parasites are grouped into distinct populations prior to estimating these parameters. Our analysis focused on using genetic relatedness and measures of spatial autocorrelation to identify population structure. Once such structure has been identified then Migrate-N could be used to determine migration rates between these different populations. Unfortunately, we did not find strong evidence for population structure among parasites in the western Kenya region, thus this analysis of gene flow may be less useful in this case. However, we have noted in the conclusion paragraph that such software is available and can be used to study gene flow, once distinct populations have been identified.**

**The statement in the conclusion paragraph reads as follows:**

**“We have also shown that directionality of parasite migration can be inferred based on genetic relatedness, and gene flow models, e.g. as implemented in migrate-n software, can be used to determine the migration rates within the region, although such models are likely to prove more useful if distinct parasite populations exist and can be identified within the region.”**
